# Evaluating the Phytochemical Contents, Antioxidant Assay, and Vitamin C Content of Selected Nutraceutical Wild Edible Plants in Northwestern Ethiopia

**DOI:** 10.1155/sci5/2274748

**Published:** 2026-05-07

**Authors:** Worku Misganaw, Getinet Masresha, Ermias Lulekal, Asmamaw Alemu, Daniel Tadesse, Paulos Getachew

**Affiliations:** ^1^ Department of Biology, College of Natural and Computational Sciences, Debark University, P.O. Box 90, Debark, Ethiopia, dku.edu.et; ^2^ Department of Biology, College of Natural and Computational Sciences, University of Gondar, P.O. Box 196, Gondar, Ethiopia, uog.edu.et; ^3^ Department of Plant Biology and Biodiversity Management, College of Natural and Computational Sciences, Addis Ababa University, P.O. Box 33658, Addis Ababa, Ethiopia, aau.edu.et; ^4^ Department of Forestry, College of Agriculture and Environmental Sciences, University of Gondar, P.O. Box 196, Gondar, Ethiopia, uog.edu.et; ^5^ Department of Plant Sciences, College of Agriculture and Environmental Sciences, University of Gondar, P.O. Box 196, Gondar, Ethiopia, uog.edu.et; ^6^ Center for Food Science and Nutrition, College of Natural and Computational Sciences, Addis Ababa University, P.O. Box 33658, Addis Ababa, Ethiopia, aau.edu.et

**Keywords:** ABTS, DPPH, flavonoid, FRAP, phenolics

## Abstract

Wild edible plants (WEPs) are valuable sources of secondary metabolites with potential nutraceutical benefits. This study aimed to analyze the phytochemical composition, antioxidant capacity, and vitamin C content of seven selected nutraceutical plants. The analyses were performed following AOAC standard protocols. A standard calibration curve was used to validate spectrophotometric measurements. One‐way ANOVA and Pearson’s correlation were used for analysis. The results showed that TPC, TFC, and vitamin C levels ranged from 106.61 ± 4.62 to 172.88 ± 8.76 mg GAE/100 g, 50.64 ± 5.64 to 154.16 ± 8.18 mg QE/100 g, and 11.3 ± 0.025 to 61.2 ± 0.071 mg/100 g, respectively. The average antioxidant activities of the studied WEPs, expressed as DPPH, IC_50_, ABTS, and FRAP values, ranged from 38.98% to 71.52%, 0.06 to 0.15 mg/mL, 27.67% to 61.76%, and 614.15 to 2872.94 mM Fe^2+^/g, respectively. Pearson correlation analysis revealed a strong positive relationship among the FRAP, ABTS, and DPPH assays (*r* = 0.93–0.99, *p* < 0.001), indicating consistent redox‐based antioxidant responses across the different methods. In conclusion, this study identifies selected WEPs as valuable sources of bioactive compounds that offer significant nutritional and health benefits, particularly in combating oxidative stress‐related disorders. These plants provide affordable, natural alternatives to synthetic antioxidants, especially in resource‐limited communities. Promoting their sustainable use and conservation can enhance dietary diversity and local resilience, highlighting their potential as crucial components of sustainable food systems in Ethiopia and beyond.

## 1. Introduction

The increasing global prevalence of malnutrition and noncommunicable diseases has intensified the search for sustainable and alternative food sources [1]. This pursuit is grounded in the “Food‐Mediator” paradigm, which posits that dietary components can directly modulate human metabolic pathways to prevent diseases [2, 3]. Wild edible plants (WEPs) are central to this paradigm and serve as reservoirs of evolutionarily optimized bioactive phytochemicals and secondary metabolites, originally produced for plant defense but also conferring significant human health benefits [3]. Building on this concept, many WEPs have been recognized for their wide range of therapeutic effects, underscoring their importance in both traditional and modern health systems. Such species are often referred to as nutraceutical plants because of their combined nutritional and medicinal attributes.

Within this broad category of health‐promoting compounds, antioxidants such as phenolics, flavonoids, and vitamin C are particularly important because they mitigate oxidative stress, a pathological condition arising from an imbalance between reactive oxygen species (ROS) and the body’s antioxidant defenses [4–6]. When ROS levels exceed the capacity of antioxidants to neutralize them, oxidative damage occurs, targeting proteins, lipids, carbohydrates, and DNA. Antioxidants counteract these effects by donating or accepting electrons, thereby terminating chain reactions and protecting cellular components [7]. The body maintains redox homeostasis through enzymatic antioxidants, such as superoxide dismutase, catalase, and glutathione peroxidase, as well as nonenzymatic antioxidants, including phenolics, flavonoids, and vitamins C and E. By complementing these endogenous defenses, dietary antioxidants derived from WEPs contribute significantly to health promotion and disease prevention [8, 9].

In addition to their antioxidant properties, phytochemicals in WEPs exhibit a wide range of biological activities, including anti‐inflammatory, anticancer, antidiabetic, antiobesity, antiarthritis, antistroke, antirespiratory, anti‐immune deficiency, and antiaging [10, 11]. These multifunctional bioactivities complement the antioxidant defenses described above, highlighting the broad role of WEPs in maintaining cellular health and preventing chronic diseases. Consequently, the inclusion of WEPs in the diet should be viewed not only as a response to food scarcity but also as a strategic approach to enhance nutritional quality and support a health‐resilient food system [12].

The Addi Arkay District in northwestern Ethiopia is widely recognized for its rich ethnobotanical knowledge and long‐standing reliance on traditional medicine and WEPs, particularly during periods of food scarcity [13]. Consistent with this background, our ethnobotanical survey documented 42 WEP species commonly utilized by local communities, highlighting the continued importance of these plants for subsistence and nutritional resilience. However, despite their traditional significance, scientific evidence regarding the phytochemical composition, antioxidant capacity, and vitamin C content of these species remains limited, underscoring the need for systematic investigation.

Therefore, the rationale for this study is to bridge this knowledge gap by providing empirical data on the nutraceutical potential of these underutilized species. The specific objectives were: (1) to quantify the total phenolic content (TPC), total flavonoid content (TFC), and vitamin C contents of seven selected WEPs; (2) to evaluate their antioxidant activity using DPPH, ABTS, and ferric reducing antioxidant power (FRAP) assays; and (3) to determine the correlations between phytochemical constituents and antioxidant capacities. This study therefore aims to conduct a comprehensive evaluation of phytochemical contents, antioxidant activity, and vitamin C levels in seven selected WEPs (*Capparis tomentosa*, *Dioscorea hispida*, *Gardenia ternifolia*, *Grewia ferruginea*, *Phoenix reclinata*, *Searsia glutinosa*, and *Ximenia americana*). The findings are expected to provide empirical evidence for identifying nutraceutically promising species, thereby supporting their safe and sustainable integration into local food systems, strengthening dietary diversification, and contributing to improved nutritional resilience and community health security.

## 2. Materials and Methods

### 2.1. Study Sites, Species Selection, Collection, and Preparation

**FIGURE 1:**
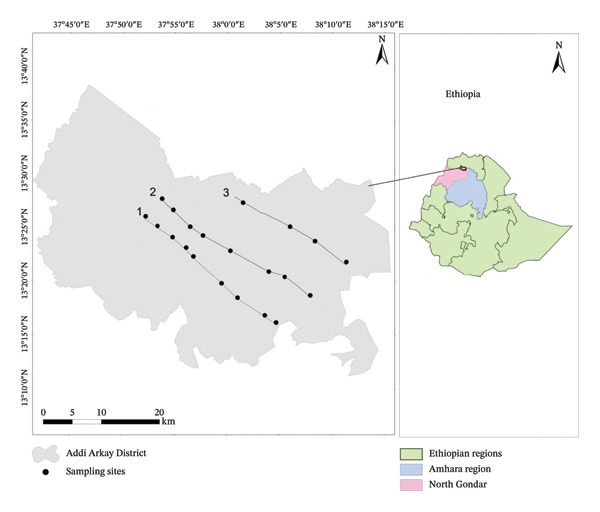
Map of the study district and sampling sites.

**FIGURE 2:**
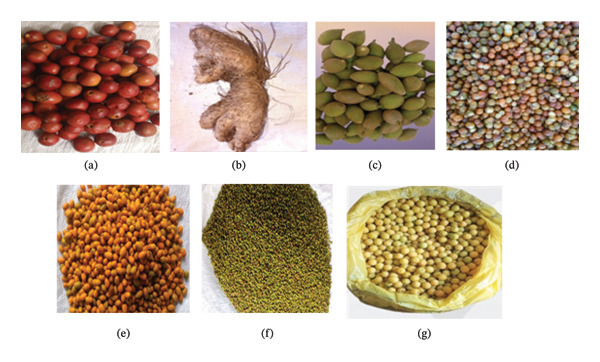
The studied nutraceutical plants. (a) *C. tomentosa*. (b) *D. hispida*. (c) *G. ternifolia*. (d) *G. ferruginea*. (e) *P. reclinata*. (f) *S. glutinosa*. (g) *X. americana*.

Seven WEP species were selected from the 42 species documented during a prior ethnobotanical survey in Addi Arkay District, North Gondar Zone, Amhara Region, northwestern Ethiopia (13°10′00″–13°40′00″ N; 37°40′00″–38°20′00″ E), with sampling locations shown in Figure 1. Species selection was based on community preference ranking, reported nutraceutical relevance, and limited available literature on phytochemical contents, antioxidant activity, and vitamin C composition (Table 1). The edible parts of each species were harvested at the peak stage of edibility, as determined through indigenous knowledge and visual maturity indicators (Figure 2). To ensure taxonomic accuracy, voucher specimens of all selected plants were collected and authenticated by a botanist at the University of Gondar.

**TABLE 1:** Nutraceutical WEPs.

Scientific name	Local name	Family	Edible part	Voucher number	References
*Capparis tomentosa* Lam.	Gimero	Capparaceae	Fruit	WM014/25	[14, 15]
*Dioscorea hispida* Dennst.	Tabile	Dioscoreaceae	Tuber	MW117/25	[16]
*Gardenia ternifolia* Schumach. & Thonn.	Gambilo	Rubiaceae	Fruit	WM005/25	[17, 18]
*Grewia ferruginea* Hochst. ex A.Rich.	Lenkwata	Malvaceae	Fruit	WM028/25	[19, 20]
*Phoenix reclinata* Jacq.	Selen	Arecaceae	Fruit	MW132/25	[21, 22]
*Searsia glutinosa* (Hochst. ex A.Rich.) Moffett	Kamuna	Anacardiaceae	Fruit	MW128/25	[23, 24]
*Ximenia americana* L.	Enkoy	Olacaceae	Fruit	WM058/25	[17, 25]

**FIGURE 3:**
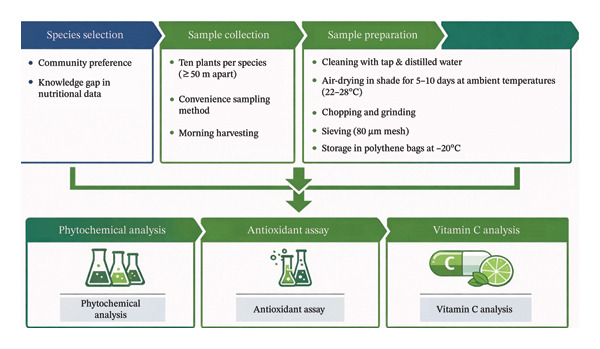
Schematic diagram of the study workflow.

Sample collection and preparation followed Roy et al. [26], with minor modifications, and the overall workflow is illustrated in Figure 3. Systematic sampling was conducted during the main harvest season (May–August 2024) based on accessibility and availability of mature edible materials. For each species, samples were collected from three independent sites, with a minimum of ten healthy, mature individuals per site, spaced ≥ 50 m apart to minimize spatial autocorrelation.

Plant materials from all sites were pooled to form approximately 2 kg of fresh composite sample per species, transported in polyethylene bags, and washed thoroughly with tap water followed by distilled water. Samples were shade‐dried for 5–10 days at ambient temperature (22°C–28°C) to minimize degradation of phytochemical constituents, antioxidant activity, and vitamin C. The dried materials were chopped, ground, and sieved through a 180‐μm mesh. The resulting powders were sealed in airtight polyethylene bags and stored at −20°C in the Botany Laboratory, University of Gondar, preserved until further analysis.

Laboratory analyses were conducted at the Food Science and Nutrition Center of Addis Ababa University, while vitamin C determination was performed at the Food Science and Nutrition Laboratory of the Ethiopian Public Health Institute. Each composite sample was analyzed in triplicate on a dry weight basis to obtain representative species‐level profiles.

For extract preparation, 5 g of powdered sample was homogenized with 50 mL of methanol and macerated for 24 h on a mechanical shaker at room temperature. The mixtures were filtered through Whatman No. 1 filter paper (Whatman International Ltd., Maidstone, England), and the solvent was removed by evaporation under a fume hood at ambient temperature. The resulting crude extracts were preserved for subsequent phytochemical, antioxidant, and vitamin C analyses.

### 2.2. Chemical Reagents and Solvents

Sodium tungstate dihydrate (Na_2_WO_4_·2H_2_O), sodium molybdate (Na_2_MoO_4_), concentrated hydrochloric acid (HCl), phosphoric acid (H_3_PO_4_), lithium sulfate monohydrate (Li_2_SO_4_·H_2_O), sodium nitrite (NaNO_2_), aluminum chloride hexahydrate (AlCl_3_·6H_2_O), sodium hydroxide (NaOH), methanol, (+)‐catechin hydrate, 2,2‐diphenyl‐1‐picrylhydrazyl (DPPH), ascorbic acid (vitamin C), sodium acetate trihydrate (C_2_H_3_NaO_2_·3H_2_O), 2,4,6‐tripyridyl‐s‐triazine (TPTZ), ferric chloride hexahydrate (FeCl_3_·6H_2_O), iron(II) sulfate (FeSO_4_), 2,2′‐azino‐bis(3‐ethylbenzothiazoline‐6‐sulfonic acid (ABTS), potassium persulfate (K_2_S_2_O_8_), Trolox (6‐hydroxy‐2,5,7,8‐tetramethylchroman‐2‐carboxylic acid), sodium chloride (NaCl), trichloroacetic acid (TCA), saturated bromine solution, thiourea solution, 2,4‐dinitrophenylhydrazine (2,4‐DNPH), sulfuric acid (H_2_SO_4_), and distilled water.

### 2.3. Phytochemical Contents of WEPs

#### 2.3.1. TPC

The TPC of the dried methanol extract, prepared from freeze‐dried samples, was determined according to the Folin–Ciocalteu assay, based on the method of [27]. The assay conditions were as follows: gallic acid standard (concentrations: 0.02–0.12 mg/mL) was added to 2.5 mL of a 10% (v/v) Folin–Ciocalteu reagent aqueous solution. After 5 min of incubation at room temperature, 2 mL of 7.5% sodium carbonate (Na_2_CO_3_) solution was added. The reaction mixture was then incubated in the dark for 30 min at 24°C. The absorbance was measured at 765 nm against a methanol blank using a Lambda 35 UV‐VIS spectrophotometer (PerkinElmer, USA). TPC was quantified from the gallic acid standard curve and expressed as mg of gallic acid equivalent per 100 g of dry weight (mg GAE/100 g DW). All analyses were performed in triplicate.

#### 2.3.2. TFC

TFC was quantified using the aluminum chloride colorimetric method described by [28]. The assay conditions were as follows: A methanolic stock solution was prepared by dissolving the dried extract (1.5 mg) in methanol (5 mL). A 30 μL aliquot of this solution was added to 3.4 mL of 30% (v/v) aqueous methanol. Subsequently, 150 μL of 0.5 M sodium nitrite (NaNO_2_) was added, followed by 150 μL of a 10% (w/v) aluminum chloride (AlCl_3_•6H_2_O) solution. After incubation at room temperature for 15 min, 2 mL of 1 M sodium hydroxide (NaOH) was added. The absorbance of the final solution was measured immediately at 765 nm against a reagent blank using a Lambda 35 UV‐VIS spectrophotometer (PerkinElmer, USA). A standard calibration curve was constructed using (+)‐catechin hydrate, and the results were expressed as mg of catechin equivalent per 100 g of dry weight (mg CE/100 g DW). All analyses were performed in triplicate.

#### 2.3.3. Vitamin C Content

Vitamin C content was determined spectrophotometrically according to the official method [29]. Briefly, 5 g of the sample was homogenized in 100 mL of 6% TCA and filtered. The filtrate was treated with bromine water and aerated. A 10 mL aliquot was mixed with 10 mL of 2% thiourea, and 4 mL of this mixture was reacted with 2,4‐DNPH at 37°C for 3 h. After cooling, 85% sulfuric acid was added and the solution was incubated in the dark for 30 min. The absorbance was measured at 515 nm using a spectrophotometer (Model 220 UV‐VIS). The experiment was performed in triplicate. Vitamin C concentration was calculated using the following formula:

(1) vitamin C mg100g=As−AbAstd−Abstd×10,

where *A*_*s*_ is the sample absorbance, *A*_*b*_ is the blank sample absorbance, *A*_std_ is the standard absorbance, and *A*_bstd_ is the blank standard absorbance.

### 2.4. Antioxidant Activity of WEPs

#### 2.4.1. DPPH Radical Scavenging Activity

The DPPH free radical scavenging activity of the samples was determined using a method adapted from [30]. Briefly, 1 mL of the methanolic extract at various concentrations (0.02–0.2 mg/mL) was mixed with 3 mL of a 0.1 mM DPPH methanolic solution. The reaction mixture was incubated in the dark for 30 min, and absorbance was measured at 517 nm using a Lambda 35 UV‐VIS spectrophotometer (PerkinElmer, USA). A blank was prepared with 1 mL of methanol and 3 mL of the DPPH solution. Radical scavenging activity was calculated as the percentage of inhibition using the following formula:

(2) % inhibition=A_blank−A_sampleA_blank×100,

where *A*_blank is the absorbance of the control and *A*_sample is the absorbance of the test sample. The results were compared to those of an ascorbic acid standard, and the half‐maximal inhibitory concentration (IC_50_) was determined from a plot of the inhibition percentage against the extract concentration.

#### 2.4.2. ABTS Radical Scavenging Activity

The ABTS radical scavenging activity was determined following the method described by [31]. Equal volumes of 7.4 mM ABTS and 2.6 mM potassium persulfate were mixed and allowed to react for 12 h in the dark to generate the ABTS^+^ working solution. This solution was then diluted with methanol to achieve an absorbance of approximately 1.17 at 734 nm. For the assay, 150 μL of each plant extract (0.01–0.15 mg/mL) was mixed with 2850 μL of the fresh ABTS^+^ solution and incubated for 2 h in the dark. The decrease in absorbance at 734 nm was measured using a Lambda 35 UV–VIS spectrophotometer (PerkinElmer, USA) against a blank and control. A Trolox standard curve (0.01–0.15 mg/mL) was used for quantification, and the results were expressed as μM Trolox equivalents (TEs) per gram of dry mass. Radical scavenging activity was calculated as the percentage of inhibition using the following formula:

(3) % inhibition=A_blank−A_sampleA_blank×100,

where *A*_blank is the absorbance of the ABTS^+^ working solution (control) and *A*_sample is the absorbance of the test sample.

#### 2.4.3. FRAP Assay

FRAP assay was performed according to the method described by [32]. The FRAP reagent was prepared by mixing 2.5 mL of 10 mM TPTZ (in 40 mM HCl), 25 mL of 300 mM acetate buffer (pH 3.6), and 2.5 mL of 20 mM FeCl_3_·6H_2_O. This reagent was incubated at 37°C for 15 min. For the assay, 150 μL of the methanolic plant extract at various concentrations (200–1000 μg/mL) was added to 1.5 mL of freshly prepared warm FRAP reagent. The mixture was incubated in the dark for 30 min, and the absorbance was measured at 593 nm. A standard curve was constructed using Trolox at concentrations ranging from 0.01 to 0.15 mg/mL. The results were expressed in FRAP units as millimolar of Fe^2+^ per g of extract (mM Fe^2+^/g extract), calculated from the linear regression of the standard curve.

### 2.5. Statistical Analysis

Statistical analysis was conducted using IBM SPSS Statistics 25.0 [33], with data reported as mean ± standard deviation. One‐way ANOVA was applied, followed by Tukey’s HSD test (*p* < 0.05) for post hoc comparisons. Pearson correlation analysis was used to evaluate the relationships between phytochemical content (total phenolics and flavonoids) and antioxidant activity (total phenolics, flavonoids, and vitamin C). The correlation coefficients (*r*) were calculated.

## 3. Results and Discussion

### 3.1. Phytochemical Contents

#### 3.1.1. TPC

**FIGURE 4:**
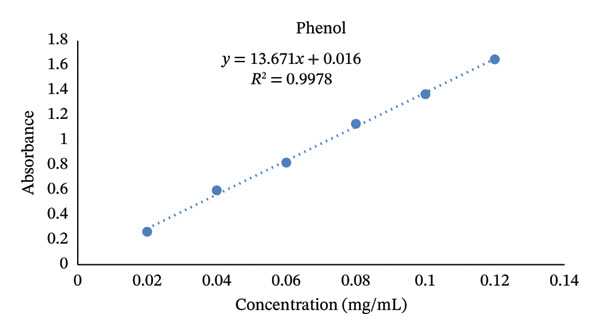
Standard curve of gallic acid at different concentrations.

The standard calibration curve for TPC showed a strong linear relationship between absorbance and concentration (*y* = 13.671*x* + 0.016, *R* ^2^ = 0.9978), indicating an excellent correlation and analytical precision (Figure 4). This high coefficient of determination (*R* ^2^) confirmed the reliability of the spectrophotometric method used to quantify the total phenolics. Different concentrations of gallic acid standards were used to validate the accuracy and reproducibility of the assay.

The TPC of the selected WEPs ranged from 106.61 ± 4.62 mg GAE/100 g in *Capparis tomentosa* to 172.88 ± 8.76 mg GAE/100 g in *Ximenia americana* on a dry weight basis (Table 2). Significant interspecies variation was observed (*p* < 0.05), which was attributed to intrinsic genetic diversity, as well as physiological and environmental factors influencing phenolic biosynthesis and accumulation [34]. Previous studies have reported a wide range of TPC in WEPs. For example, in northeastern Ethiopia, TPC was low, ranging from 12.69 ± 0.00 mg/100 g in *Amaranthus hybridus* grain to 17.02 ± 0.03 mg/100 g in *Haplocarpha rueppelii* leaf [35], whereas in northern Uganda, TPC was much higher, from 1339 ± 0.26 mg/100 g in *Acalypha rhomboidea* to 6425 ± 0.54 mg/100 g in *Ipomoea eriocarpa* [4]. Phenolic compounds contribute to the quality and nutritional value of plants by affecting color, taste, aroma, and flavor, while also providing health benefits and protecting plants from oxidative stress and damage by microorganisms, insects, and herbivores [36]. The elevated phenolic content in *X. americana* indicates a strong antioxidant potential, contributing to free radical scavenging and the prevention of oxidative stress–related chronic diseases. Although no upper safe limit for phenolic intake has been established, moderate consumption through natural dietary sources is considered beneficial due to their well‐documented antioxidant and anti‐inflammatory properties [34].

**TABLE 2:** Phytochemical composition of selected WEPs.

WEPs	TPC (mg GAE/100 g)	TFC (mg QE/100 g)	Vit. C (mg/100 g)
*Capparis tomentosa*	106.61 ± 4.62^g^	50.64 ± 5.64^g^	45.0 ± 0.18^d^
*Dioscorea hispida*	156.30 ± 9.33^c^	91.15 ± 0.27^d^	32.5 ± 0.08^e^
*Gardenia ternifolia*	135.10 ± 2.82^e^	53.06 ± 0.43^f^	11.3 ± 0.025^g^
*Grewia ferruginea*	136.95 ± 2.36^d^	69.59 ± 2.23^e^	24.6 ± 0.22^f^
*Phoenix reclinata*	118.93 ± 1.67^f^	94.11 ± 3.31^c^	61.2 ± 0.071^a^
*Searsia glutinosa*	163.34 ± 5.23^b^	154.16 ± 8.18^a^	47.0 ± 0.22^c^
*Ximenia americana*	172.88 ± 8.76^a^	152.20 ± 1.68^b^	52.5 ± 0.32^b^

*Note:* Data are presented as mean ± SD on a dry weight basis (DW, mg/100 g). Different superscript letters within a column indicate statistically significant differences (*p* < 0.05).

#### 3.1.2. TFC

**FIGURE 5:**
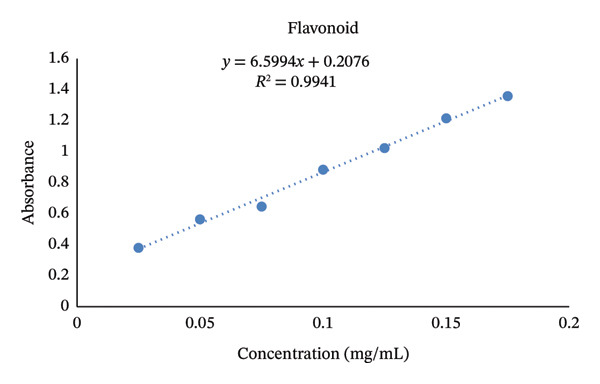
Standard curve of quercetin at different concentrations.

The standard calibration curve for TFC exhibited a strong linear relationship between the absorbance and concentration (*y* = 6.5994*x* + 0.2076, *R* ^2^ = 0.9941), as shown in Figure 5. The high coefficient of determination (*R* ^2^) indicated excellent linearity, suggesting that the method provides accurate and reliable quantification of flavonoid content within the tested range. Quercetin standards at varying concentrations were used to validate spectrophotometric measurements, ensuring the precision and reproducibility of the assay.

TFC of the analyzed WEPs ranged from 50.64 ± 5.64 mg QE/100 g for *Capparis tomentosa* to 154.16 ± 8.18 mg QE/100 g for *Searsia glutinosa* (Table 2). Comparable findings have been reported in WEPs, where TFC contents ranged from 79.70–112.85 mg CE/100 g [37] and from 45.24–178.46 mg RE/100 g [38]. The elevated flavonoid concentrations observed in *S. glutinosa* and *X. americana* could be due to adaptive biochemical defense mechanisms against oxidative stress, a common trait among plants thriving in arid and semiarid environments [39]. Flavonoids play crucial roles as potent antioxidants and anti‐inflammatory agents, enhancing the overall nutraceutical and therapeutic potential of these species [40]. Although no established upper dietary limit exists, epidemiological studies indicate that a daily flavonoid intake of 200–500 mg is associated with reduced risks of cardiovascular and metabolic diseases [41]. Therefore, WEPs could make a valuable contribution to dietary antioxidant intake, particularly in resource‐limited communities with restricted access to conventional fruits and vegetables.

#### 3.1.3. Vitamin C (Ascorbic Acid)

**FIGURE 6:**
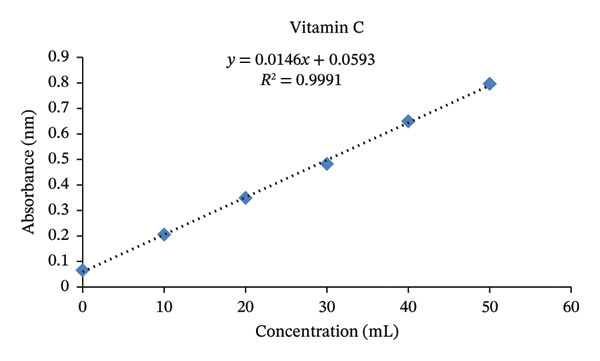
Standard curve of ascorbic acid at different concentrations.

The standard calibration curve for Vitamin C showed an excellent linear relationship between absorbance and concentration (*y* = 0.0146*x* + 0.0593, *R* ^2^ = 0.9991) (Figure 6). The high correlation coefficient indicated outstanding linearity, confirming the precision and reliability of the spectrophotometric method for ascorbic acid quantification. Standard solutions of pure ascorbic acid at varying concentrations were used to construct the calibration curve, which served to validate the analytical accuracy and reproducibility of the assay in determining vitamin C levels in food samples.

The vitamin C content of the studied WEPs ranged from 11.3 ± 0.025 mg/100 g in *Gardenia ternifolia* to 61.2 ± 0.071 mg/100 g in *Phoenix reclinata* (Table 2). Most species, including *Ximenia americana* (52.5 ± 0.32 mg/100 g) and *Searsia glutinosa* (47.0 ± 0.22 mg/100 g), also contained substantial vitamin C levels, while *Gardenia ternifolia* had the lowest content. These values were higher than those reported for WEPs from the North Shoa Zone, Oromia region (0.215–1.612 mg/100 g) [42], and comparable to the range documented for WEPs from Lasta District, Northeastern Ethiopia (2.16–70.42 mg/100 g) [43]. Variation in vitamin C content among species may be due to genetic differences, environmental conditions, plant parts analyzed, and postharvest degradation, as vitamin C is highly sensitive to light, heat, and storage [44]. While most WEPs alone cannot meet the recommended dietary allowance (RDA) of 60 mg/day for adults, *Phoenix reclinata* approaches this requirement, and regular consumption of these species can provide valuable dietary antioxidants. This is particularly important in rural areas where vitamin‐rich fruits are scarce and populations are at risk of deficiencies such as scurvy [43]. Thus, these WEPs serve as important supplementary sources of vitamin C within diversified diets [35].

### 3.2. Antioxidants Scavenging Activities of WEPs

In this study, the antioxidant capacities of the selected WEPs were evaluated using DPPH, ABTS, and FRAP assays to assess their potential health‐promoting properties. These complementary methods provide a comprehensive understanding of the ability of plants to donate electrons or hydrogen atoms, neutralize free radicals, and prevent oxidative damage.

#### 3.2.1. DPPH Radical Scavenging Activity

**FIGURE 7:**
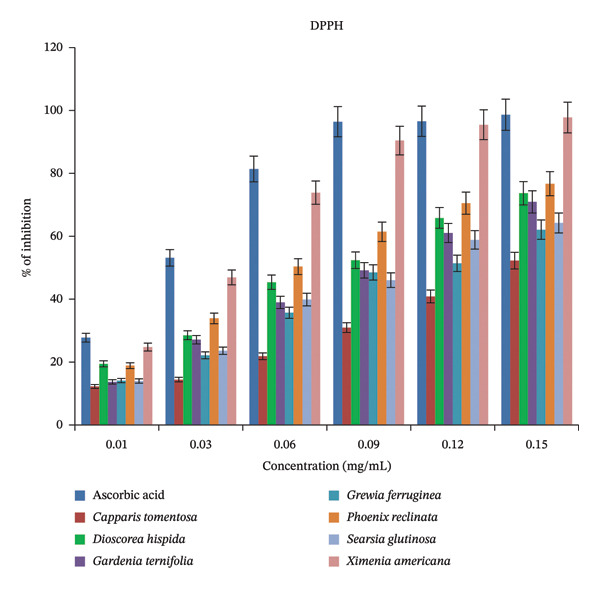
DPPH radical scavenging activity as % of inhibition at various concentrations (mg/mL).

The antioxidants present in the plant extracts interacted with DPPH, leading to a reduction in its deep violet color to a stable yellow form through the donation of electrons or hydrogen atoms. This rapid reaction caused a marked decrease in the absorbance at 517 nm, indicating the scavenging activity of the extracts. As illustrated in Figure 7, at a concentration of 0.15 mg/mL, the DPPH scavenging activity of the *Ximenia americana* extract reached 97.63%, which was comparable to that of the ascorbic acid standard (97.44%). This concentration‐dependent trend is consistent with previous findings [6, 35], which reported a similar pattern.

The average DPPH scavenging activities of methanolic extracts of the seven WEPs are summarized in Table 3, showing mean inhibition values ranging from 38.98% to 71.52%. Among the tested plants, *Ximenia americana* exhibited the highest DPPH radical scavenging activity (71.52%), followed by *Dioscorea hispida* (62.20%) and *Phoenix reclinata* (51.95%), whereas *Grewia ferruginea* showed the lowest activity (38.98%). Comparable findings were reported by [6], where DPPH scavenging activity ranged from 50.09% in *Dioscorea praehensilis* tuber to 87.63% in *Solanum nigrum* leaf among five WEPs from Southwest Ethiopia, and by [45], who observed values ranging from 18.82% in *Cardamine hirsuta* to 53.74% in *Melothria perpusilla* among six WEPs consumed by the Bodos of Northeast India. The observed differences in scavenging activity among the studied WEPs could be attributed to their genetic variation, environmental growth conditions, and differences in the types and quantities of the bioactive compounds present.

**TABLE 3:** Antioxidant activity of seven WEPs.

Scientific name	DPPH % of inhibition	IC_50_ mg/mL	ABTS % of inhibition	FRAP (mM Fe^2+^/g)
*Capparis tomentosa* Lam.	47.52	0.08	49.48	2023.13
*Dioscorea hispida* Dennst.	62.20	0.07	55.99	2609.51
*Gardenia ternifolia* Schumach. & Thonn.	43.47	0.13	32.68	763.20
*Grewia ferruginea* Hochst. ex A.Rich.	38.98	0.15	27.67	614.15
*Phoenix reclinata* Jacq.	51.95	0.09	43.27	1700.77
*Searsia glutinosa* (Hochst. ex A.Rich.) Moffett	41.08	0.12	35.97	1173.28
*Ximenia americana* L	71.52	0.06	61.76	2872.94
Ascorbic acid	75.63	0.03	—	—
Trolox	—	0.05	64.09	1533.36

The DPPH assay was further expressed in terms of IC_50_. The IC_50_ value is defined as the concentration of the crude extract required to scavenge 50% of ROS or inhibit the oxidation process by 50% [46]. This was calculated from the concentration–inhibition activity curve. Since IC_50_ is inversely related to antioxidant capacity, a lower IC_50_ value indicates a stronger antioxidant activity. According to established classifications, antioxidant strength is considered very strong when IC_50_ < 0.05 mg/mL, strong between 0.05 and 0.10 mg/mL, moderate between 0.10 and 0.25 mg/mL, weak between 0.25 and 0.50 mg/mL, and inactive when exceeding 0.50 mg/mL [47]. As shown in Table 3, the IC_50_ values of methanolic extracts from the seven WEPs ranged from 0.06 to 0.15 mg/mL, indicating varying levels of antioxidant potency. *Ximenia americana* (IC_50_ = 0.06 mg/mL), *Dioscorea hispida* (0.07 mg/mL), *Phoenix reclinata* (0.09 mg/mL), and *Capparis tomentosa* (0.08 mg/mL) exhibited strong antioxidant activity, while *Gardenia ternifolia* (0.13 mg/mL) and *Searsia glutinosa* (0.12 mg/mL) demonstrated moderate activity. Similarly, IC_50_ values for WEPs have been reported to range from 0.08 mg/mL in *Solanum nigrum* to 0.62 mg/mL in *Trilepisium madagascariense* [6]; from 0.13 mg/mL in *Melothria perpusilla* to 1.66 mg/mL in *Natsiatum herpeticum* in Southwest Ethiopia [45]; from 0.025 mg/mL in the leaf of *Amaranthus hybridus* to 0.197 mg/mL in its seed in Northeastern Ethiopia [35]; and from 0.14 mg/mL in *Acalypha rhomboidea* to 0.45 mg/mL in *Heterotis rotundifolia* in Northern Uganda [4].

In general, plant extracts with high DPPH radical scavenging potential exhibit strong reducing activity against oxidative stress. Therefore, the antioxidant potential of these WEPs suggests their promising role in mitigating oxidative damage, extending food shelf life, and offering health benefits associated with the prevention of oxidative stress‐related disorders. These results highlight the nutritional and therapeutic importance of the studied WEPs as potent natural antioxidants capable of enhancing health resilience and contributing to improved food and nutrition security in local communities.

#### 3.2.2. ABTS Radical Scavenging Activity

**FIGURE 8:**
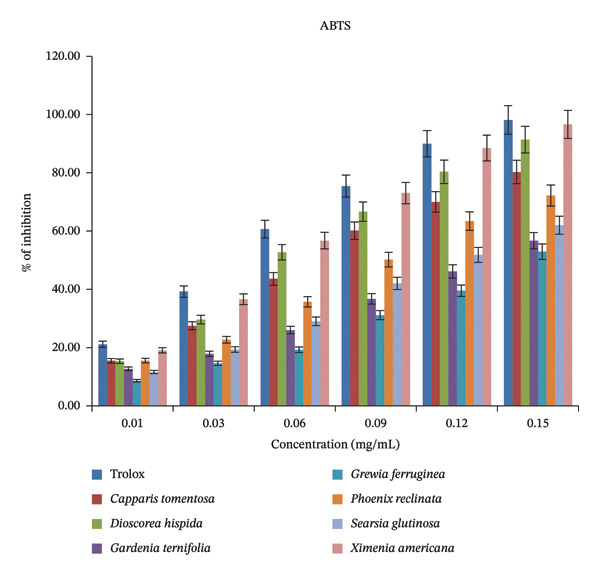
ABTS radical scavenging activity as % of inhibition at various concentrations (mg/mL).

In the ABTS•^+^ radical scavenging assay, which is based on an electron transfer mechanism, the blue‐colored ABTS radical cation (ABTS•^+^) is reduced to a colorless form by antioxidants, and the decrease in absorbance is measured spectrophotometrically [48]. The ABTS scavenging activity of the extracts increased proportionally with concentration, indicating a concentration‐dependent antioxidant response (Figure 8). Similar to the DPPH results in this study, at the highest concentration (0.15 mg/mL), *Ximenia americana* exhibited the highest radical scavenging activity (96.63%), which was comparable to that of the Trolox standard (98.12%). Other species, including *Dioscorea hispida* (91.37%), *Capparis tomentosa* (80.27%), and *Phoenix reclinata* (72.17%), also demonstrated strong ABTS scavenging potential, whereas *Gardenia ternifolia* (56.69%), *Grewia ferruginea* (52.93%), and *Searsia glutinosa* (62.02%) displayed relatively lower inhibition values.

The average ABTS radical scavenging activity of the methanolic extracts from the seven WEPs varied considerably, ranging from *Grewia ferruginea* (27.67%) to *Ximenia americana* (61.76%), demonstrating diverse antioxidant efficiencies among the species (Table 3). The strongest ABTS radical inhibition was recorded for *Ximenia americana* (61.76%), followed by *Dioscorea hispida* (55.99%) and *Capparis tomentosa* (49.48%), whereas *Gardenia ternifolia* (32.68%) exhibited the weakest activity. In comparison, a relatively higher and narrower range of ABTS inhibition from *Blumea lanceolaria* (60.71%) to *Eryngium foetidum* (69.34%) has been reported in 11 WEP species from Assam, India [49].

The strong antioxidant response observed in the present study could be associated with the presence of high‐molecular‐weight phenolic constituents that possess an enhanced capacity to neutralize ABTS radicals. Their antioxidant efficacy is influenced by factors such as molecular size, number of aromatic rings, and arrangement and type of hydroxyl groups, rather than only by the presence of specific functional groups [49, 50]. Overall, the results emphasize the nutritional and pharmacological importance of WEPs as promising sources of natural antioxidants that can reduce oxidative damage, improve food stability, and support health resilience in nutritionally vulnerable populations. These findings highlight the nutritional and therapeutic significance of the studied WEPs as potent natural antioxidants with potential dietary and pharmaceutical applications that can mitigate oxidative stress and enhance health resilience in food‐insecure communities [51].

#### 3.2.3. FRAP

**FIGURE 9:**
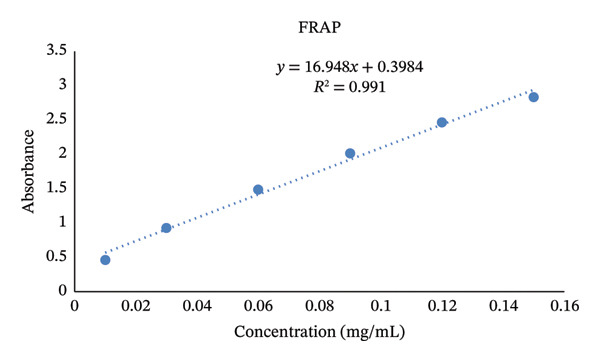
Standard curve of Trolox at different concentrations for the FRAP assay.

The standard curve of Trolox at different concentrations for the FRAP assay was calculated using the following equation: *y* = 16.948*x* + 0.3984 (*R* ^2^ = 0.991). The high coefficient of determination confirmed the accuracy, precision, and reliability of the analytical method used to evaluate the antioxidant capacities of the samples (Figure 9).

The FRAP values of the selected WEPs ranged from 614.15 to 2872.94 mM Fe^2+^/g (Table 3). Among the studied species, *Ximenia americana*, surpassing even the Trolox standard, exhibited the highest reducing capacity (2872.94 mM Fe^2+^/g), followed by *Dioscorea hispida* (260.95 mM Fe^2+^/g) and *Capparis tomentosa* (202.31 mM Fe^2+^/g), while *Grewia ferruginea* showed the lowest activity (614.15 mM Fe^2+^/g). Comparable but lower FRAP values were previously reported for methanolic extracts of *Drymaria cordata* (0.065 mM TE/g) to *Lippia javanica* (0.799 mM TE/g) [49] and for *Dioscorea praehensilis* (49.16 mM Fe^2+^/g) to *Solanum nigrum* (188.12 mM Fe^2+^/g) [6]. The high reducing activity observed in *X. americana* (2872.94 mM Fe^2+^/g) and *D. hispida* (2609.51 mM Fe^2+^/g) is substantially greater than values reported in similar studies, which may be attributed to their exceptionally high phenolic content (172.88 and 156.30 mg GAE/100 g, respectively) and the presence of specific high‐molecular‐weight phenolic polymers with enhanced electron‐donating capacity. The high reducing activity observed in these WEPs may be attributed to their richness in phenolic and other antioxidant compounds, which are capable of donating electrons to neutralize free radicals. The FRAP assay measures the ability of a sample to reduce ferric (Fe^3+^) to ferrous (Fe^2+^) ions at 593 nm, thereby reflecting the total antioxidant potential of the extracts [52]. The strong reducing power exhibited by these plants suggests their potential as functional foods for mitigating oxidative stress–related disorders such as cardiovascular diseases and diabetes [53].

### 3.3. Correlation

The correlation coefficients (*r*) were interpreted as very weak (0.00–0.19), weak (0.20–0.39), moderate (0.40–0.59), strong (0.60–0.79), and very strong (0.80–1.00) [6]. Pearson correlation analysis (Table 4) revealed a very strong positive correlation among the antioxidant assays FRAP, ABTS, and DPPH (*r* = 0.93–0.99, *p* < 0.001), indicating that these methods measure antioxidant potential through similar redox‐based mechanisms. This strong interassay relationship is consistent with previous studies on leafy and WEPs that reported comparable patterns [54]. TPC showed a moderate positive correlation with FRAP (*r* = 0.45), ABTS (*r* = 0.41), and DPPH (*r* = 0.55). However, these correlations were not statistically significant (*p* > 0.05 for all), suggesting a trend but not definitive proof that phenolic compounds are the primary drivers of antioxidant activity in this specific dataset. While phenolic compounds are known to play a central role in antioxidant activity [55], the lack of statistical significance here may be due to the limited sample size (*n* = 7) or the influence of other nonphenolic antioxidants.

**TABLE 4:** Correlation matrix.

	TPC	TFC	Vit. C	FRAP	ABTS	DPPH
TPC	1					
TFC	0.31 (0.49)	1				
Vit. C	0.40 (0.38)	−0.04 (0.93)	1			
FRAP	0.45 (0.32)	−0.34 (0.45)	0.47 (0.28)	1		
ABTS	0.41 (0.36)	−0.32 (0.49)	0.45 (0.32)	0.996^∗∗∗∗^ (*p* < 0.001)	1	
DPP	0.55 (0.20)	−0.16 (0.73)	0.65 (0.11)	0.926^∗∗∗∗^ (*p* < 0.001)	0.930^∗∗∗∗^ (*p* < 0.001)	1

^∗∗∗^Correlation is significant at the 0.01 level (2‐tailed).

Vitamin C also showed moderate to strong positive correlations with FRAP (*r* = 0.47), ABTS (*r* = 0.45), and DPPH (*r* = 0.65), but these were also not statistically significant (*p* > 0.05 for all), highlighting its complementary contribution to overall antioxidant activity. The close association between vitamin C and phenolic content suggests their synergistic role in scavenging free radicals and enhancing the redox balance. In contrast, TFC displayed weak negative correlations with FRAP (*r* = −0.34, *p* = 0.45), ABTS (*r* = −0.32, *p* = 0.49), and DPPH (*r* = −0.16, *p* = 0.73) assays, implying a limited influence on the overall antioxidant performance of the extracts [4]. Collectively, these findings indicate that phenolic compounds and vitamin C were the primary contributors, the correlations were not statistically significant, and thus, no definitive conclusions about their primary roles can be drawn from this study regarding the antioxidant potential of the studied WEPs. The high agreement among FRAP, ABTS, and DPPH assays confirmed the consistency and reliability of the antioxidant evaluation methods used.

## 4. Conclusion and Recommendations

This study revealed that selected WEPs from northwestern Ethiopia are valuable sources of bioactive compounds with significant nutritional and health‐promoting potential. These plants, containing varying levels of phenolics, flavonoids, and vitamin C, play a crucial role in neutralizing free radicals and preventing oxidative stress‐related disorders such as cardiovascular diseases, diabetes, and certain cancers. The considerable variation in phytochemical and antioxidant levels among species underscores the influence of genetic, environmental, and physiological factors on their biochemical compositions. Furthermore, the relatively high concentrations of these beneficial compounds suggest that these plants could serve as affordable natural alternatives to synthetic antioxidants, particularly in food‐insecure and resource‐limited communities.

The moderate positive associations of TPC and vitamin C with antioxidant assays were not statistically significant, preventing definitive conclusions about their primary roles. However, the very strong and significant correlations among the FRAP, ABTS, and DPPH assays validated the reliability of the antioxidant methods employed. Promoting sustainable utilization and conservation of these indigenous species can contribute to improved dietary diversity, community nutrition, and local economic resilience. Overall, this research supports the potential of WEPs as underexplored, yet valuable components of sustainable food systems in Ethiopia and beyond.

### 4.1. Limitations of the Study

This study was limited to a specific geographic area and focused on samples analyzed on a dry weight basis, which may not capture variations in phytochemical composition between fresh and dried samples across different agroecologies. Additionally, antioxidant assays were conducted *in vitro* and may not fully represent *in vivo* antioxidant activity. Furthermore, the correlation analysis between phytochemicals and antioxidant activity lacked statistical significance, likely due to the small sample size (*n* = 7), and future studies with a larger number of species are needed to clarify these relationships.

## Author Contributions

Worku Misganaw: conceptualization, methodology, investigation, formal analysis, visualization, software, writing–original draft, and writing–reviewing and editing. Getinet Masresha: supervision, validation, fund acquisition, and project administration. Ermias Lulekal: supervision and validation. Asmamaw Alemu: supervision, validation, fund acquisition, and project administration. Daniel Tadesse: conceptualization, data curation, supervision, and writing–reviewing and editing. Paulos Getachew: data curation and provision of required laboratory equipment and reagents.

## Funding

Data collection for this research was financially supported by the University of Gondar (Grant No. RCSTT 02), and field equipment was provided by Idea Wild​ (501(c)(3)).

## Ethics Statement

This study did not involve human participants or animal experimentation. Prior to the field collection of plant specimens, official permission was obtained from the Addi Arkay District Offices of Agriculture and Environmental Protection. To ensure ethical conduct and sustainability, plant sampling was carried out in strict accordance with the guidelines of the International Union for Conservation of Nature and the Convention on International Trade in Endangered Species of Wild Fauna and Flora.

## Conflicts of Interest

The authors declare no conflicts of interest.

## Data Availability

The data that support the findings of this study are available from the corresponding author upon reasonable request.
